# Spatial Mnemonic Encoding: Theta Power Decreases and Medial Temporal Lobe BOLD Increases Co-Occur during the Usage of the Method of Loci

**DOI:** 10.1523/ENEURO.0184-16.2016

**Published:** 2017-01-10

**Authors:** Marie-Christin Fellner, Gregor Volberg, Maria Wimber, Markus Goldhacker, Mark W. Greenlee, Simon Hanslmayr

**Affiliations:** 1 Department of Psychology, University of Konstanz, 78457 Konstanz, Germany; 2 Department of Neuropsychology, Institute of Cognitive Neuroscience, Ruhr University Bochum, 44801 Bochum, Germany; 3 Institute of Experimental Psychology, University of Regensburg, 93040 Regensburg, Germany; 4 School of Psychology, University of Birmingham, Birmingham B15 2TT, United Kingdom

**Keywords:** EEG, fMRI, memory encoding, method of loci, mnemonics, theta oscillations

## Abstract

The method of loci is one, if not the most, efficient mnemonic encoding strategy. This spatial mnemonic combines the core cognitive processes commonly linked to medial temporal lobe (MTL) activity: spatial and associative memory processes. During such processes, fMRI studies consistently demonstrate MTL activity, while electrophysiological studies have emphasized the important role of theta oscillations (3–8 Hz) in the MTL. However, it is still unknown whether increases or decreases in theta power co-occur with increased BOLD signal in the MTL during memory encoding. To investigate this question, we recorded EEG and fMRI separately, while human participants used the spatial method of loci or the pegword method, a similarly associative but nonspatial mnemonic. The more effective spatial mnemonic induced a pronounced theta power decrease source localized to the left MTL compared with the nonspatial associative mnemonic strategy. This effect was mirrored by BOLD signal increases in the MTL. Successful encoding, irrespective of the strategy used, elicited decreases in left temporal theta power and increases in MTL BOLD activity. This pattern of results suggests a negative relationship between theta power and BOLD signal changes in the MTL during memory encoding and spatial processing. The findings extend the well known negative relation of alpha/beta oscillations and BOLD signals in the cortex to theta oscillations in the MTL.

## Significance Statement

Studies investigating the oscillatory correlates of memory encoding largely focus on activity in the theta frequency and often implicitly assume that increases in theta activity reflect similar processes as typically reported increased medial temporal lobe (MTL) activity in fMRI studies. The presented study found decreases, not increases, in theta power closely mapping to MTL BOLD increases in the exact same paradigm. The reported findings importantly contribute to the question of how and which oscillatory activity indexes MTL memory processes. This finding is in line with studies showing a negative relationship between low-frequency power and BOLD changes in the cortex, and challenges the assumption that theta power increases reflect MTL activity.

## Introduction

Converging work in animals and humans has linked two important cognitive functions to medial temporal lobe (MTL) structures: spatial processing and memory (Burgess et al., 2002; Buzsáki and Moser, 2013). The influence of spatial processing on memory formation has been documented since ancient Greek times: the method of loci, a mnemonic strategy based on linking to-be-learned material to waypoints on a familiar route is an outstandingly efficient strategy to memorize new information (Roediger, 1980). This spatial mnemonic is up to the present day the preferred strategy of memory athletes memorizing impressive amounts of arbitrary information by associating it with spatial cues (Maguire et al., 2003).

Especially in animals, but also in humans, MTL theta oscillations have been implicated in spatial processing and navigation (Vanderwolf, 1969; Ekstrom et al., 2005; Watrous et al., 2013b). However, concerning memory formation, there is still an ongoing debate about the functional relationship of theta oscillations and MTL activity during memory encoding: are increases or decreases in theta power related to memory formation and MTL engagement (for review, see Hanslmayr and Staudigl, 2014)? Several studies report increases in theta power during memory formation during subsequently remembered items in contrast to subsequently forgotten items [subsequent memory effect (SME)] and hypothesized that these increases in theta power reflect MTL involvement (Klimesch et al., 1996; Nyhus and Curran, 2010; Staudigl and Hanslmayr, 2013; Backus et al., 2016). In contrast, other studies found decreased theta activity to be related to successful memory encoding (Burke et al., 2013; Long et al., 2014; Greenberg et al., 2015; Crespo-García et al., 2016). Concerning fMRI, a more consistent picture emerges where MTL structures are reliably more active during memory tasks (Kim, 2011), especially during tasks that combine associative and spatial processing (Uncapher et al., 2006; Bird and Burgess, 2008; Staresina and Davachi, 2009).

A ubiquitous finding in the cortex is a negative relation between BOLD signal and low-frequency power (1–30 Hz; Mukamel et al., 2005; Hanslmayr et al., 2011; Scheeringa et al., 2011; Hermes et al., 2014; Zumer et al., 2014). However, studies investigating theta power changes during spatial processing and associative memory formation suggest a positive relation between MTL activity and theta power (Kaplan et al., 2012; Staudigl and Hanslmayr, 2013; Backus et al., 2016). It therefore remains an open question whether increases or decreases in theta power indeed reflect increases in MTL activity (Lisman and Jensen, 2013).

**Figure 1.**
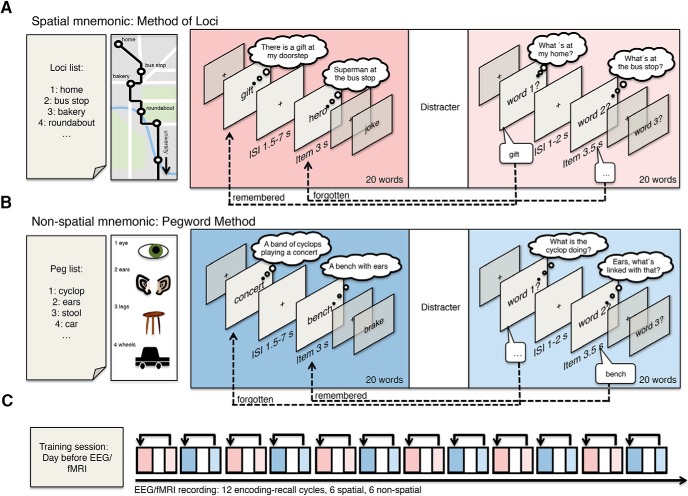
Memory encoding paradigm. ***A***, ***B***, Participants were trained to use two mnemonic encoding strategies: the spatial method of loci (***A***) and the nonspatial pegword method (***B***). In both methods, participants have to link internal cues, which are either familiar waypoints or associations of items to numbers, to items presented during the encoding phase. During each encoding phase, lists of 20 words were presented sequentially followed by a distracter task and a free recall phase. ***C***, The whole experiment entailed a training phase the day before and 12 encoding–recall cycles during EEG or fMRI recordings.

In order to investigate the relationship between MTL BOLD signals and theta power dynamics during memory encoding, we instructed participants to use associative mnemonic encoding strategies: the spatial method of loci and the nonspatial pegword method (Fig. 1). Both mnemonics entail linking to-be-learned items to internal cues, waypoints in the case of the method of loci and number-related pegs in the case of the pegword method (Fig. 1*A*,*B*). In the spatial mnemonic participants associated to-be-learned items with locations [i.e., a “hero” (item number two) waiting at a “bus stop” (the second loci cue)]. In the nonspatial mnemonic, participants linked items with number associations [i.e., a “bench” (item number 2) with “ears” (pegword number two)]. The nonspatial pegword method was specifically chosen to investigate whether SMEs during spatial processing are qualitatively different compared with nonspatial associative encoding, as it has been proposed that spatial processes might be the foundation of episodic memory processes (Buzsáki and Moser, 2013; Ekstrom, 2014; Buffalo, 2015). Consequently, spatial processing during the method of loci should lead to higher involvement of MTL regions, whereas successful encoding in both encoding tasks might similarly rely on MTL activity, irrespective of the spatial nature of the processing task. Note that EEG and fMRI were measured in separate groups of participants (Mukamel et al., 2005), because movement artifacts severely limit the interpretability of theta activity in simultaneous EEG-fMRI (Fellner et al., 2016).

## Materials and Methods

### Subjects and recording sessions

Two separate groups of participants underwent EEG recording (30 participants) and fMRI scanning (25 participants). Nine EEG datasets had to be excluded (trial numbers below 15 after artifact correction), resulting in a sample of 21 datasets (age, 18–24 years; mean age, 20.19 years; 12 females). Two fMRI datasets had to be excluded: one because of a missing structural scan and one because of memory performance 2 SDs below the average recall rate across all participants in both encoding conditions, resulting in a sample of 23 datasets (age range, 18–36 years; mean age, 22.9 years; 15 females).

All subjects spoke German as their native language, reported no history of neurologic or psychiatric disease, and had normal or corrected-to-normal vision. All participants gave their written informed consent, and the experimental protocol was approved by the local ethical review board. During fMRI, simultaneous EEG was recorded. Major MR scanner-induced EEG artifacts, especially in the theta frequency range, prevented reliable analysis of the simultaneously recorded EEG (for a discussion of these artifacts, see Fellner et al., 2016).

### Task design

A training session took place one day before fMRI or EEG recording to ensure proper usage of both mnemonics. Prior to training, participants received basic information about mnemonics and were instructed to prepare a list of loci. These loci were 20 individually chosen waypoints taken from a way highly familiar to the participants (for most of our participants, the way to the university). Additionally, participants were asked to memorize 20 pegword cues that were given in the basic experimental information. The training session then consisted of three parts: first, the 20 waypoints were checked for task suitability and memorization of all loci and pegword cues was tested. Second, participants had to encode an exemplary list of 20 words naming all associations used between words and the loci or pegword cues. If the chosen associations did not fit with the task instruction, the training session was run again. Third, four practice encoding–recall cycles with the same timing as during EEG and fMRI were performed.

The exact same paradigm was presented to participants during fMRI or EEG recordings the day after the training session. In the encoding phase, 20 to-be-learned words were presented sequentially (word presented for 3 s followed by fixation cross shown for 1.5–7 s exponentially jittered). Participants were instructed to associate each word with the corresponding cue in the loci or pegword sequence. Using the spatial method of loci, they were instructed to visualize to-be-learned items (e.g., first item gift) at the respective waypoint (e.g., the first loci cue home). For instance, participants might imagine a nicely wrapped gift at their doorstep (Fig. 1*A*). During the nonspatial pegword method, they were asked to focus on semantic relations between the item (e.g., first item concert) and the respective pegword (e.g., first pegword cyclops), such that a possible association could be “A band of cyclops playing a concert” (Fig. 1B). During recall, participants were instructed to use the pegwords and loci way points as retrieval cues to recall the words in the same sequence as during the encoding phase. Participants were asked to recall the 20 words of the prior encoding phase in their original order, whenever the screen showed a “word no. x” cue. Only words recalled in the correct relative order were included as remembered trials; that is, if a whole sequence of words was shifted (e.g., words 5–10 recalled as words 7–12), the items were counted as correctly remembered, whereas items that were recalled in the wrong relative sequence (i.e., recalling word 6 prior to word 5) were not counted as remembered. Notably, only very few items were remembered in the wrong sequence (EEG: spatial M = 1.45 words; nonspatial M = 1.59 words; fMRI: spatial M = 2.26 words; nonspatial M = 2.35 words) and the number of out-of-sequence trials did not differ between encoding conditions (EEG: *t*_(20)_ = 0.4, *p* = 0.68; fMRI: *t*_(22)_ = −0.168, *p* = 0. 87) . Items recalled in the wrong sequence were discarded from further analysis, and trials not recalled were labeled as forgotten.

The fMRI- or EEG-recorded part of the experiment consisted of 12 repeated memory-encoding and recall cycles, with each encoding–recall block consisting of an encoding phase, a visual detection task, a free recall memory test, and a short 20 s rest period (Fig. 1*C*). Each experiment was split into four consecutive recording sessions to keep file sizes manageable; the MR image acquisition was thus stopped after every third encoding–recall cycle. A visual detection task (∼2.5 min) was serving as a distracter task (a similar task as in the study by Hanslmayr et al., 2013). In-scanner verbal responses were recorded using an fMRI-compatible microphone (MRconfon). Scanner noise was removed from the resulting audio files using the free software package Audacity (http://audacity.sourceforge.net/). For two participants, data from one of the four fMRI sessions had to be discarded because of faulty microphone recordings. During the EEG experiment, recall performance was scored manually by the experimenter.

As study material, 360 words were drawn from the MRC Psycholinguistic Database (Coltheart, 2007), translated into German, and separated into 16 lists with 20 words each. Four lists were used during the training session; the other 12 lists were used during the EEG and fMRI recordings. Each of these 12 lists was matched according to average word frequency (M = 61.98 words; SEM: 1.58 words), number of letters (M = 5.59 letters; SEM: 0.04 letters), syllables (M = 1.84 syllables; SEM: 0.02 syllables), concreteness (M = 375.65; SEM: 2.17), and imageability (M = 414.21; SEM: 2.83). Word lists were counterbalanced across participants and encoding tasks. Word order in each list was randomized.

### EEG recording

The EEG was recorded from 63 channels in an equidistant montage (BrainampMR, EasyCap; RRID: SCR_009443). Recordings were referenced to Fz and later re-referenced to average reference. Impedances were kept at <20 kΩ. The signals were amplified between 0.1 and 250 Hz. The EEG data were sampled at 500 Hz with an amplitude resolution of 0.5 µV

### EEG preprocessing and analysis

All EEG data analyses were carried out using custom MATLAB scripts (RRID: SCR_001622) and fieldtrip (http://www.fieldtriptoolbox.org; RRID: SCR_004849; Oostenveld et al., 2011). Data were epoched in trials from −2.5 to 3.5 s around each item onset during encoding. Data were visually inspected to exclude trials with idiographic artifacts (channel jumps, muscle artifacts, noisy channels) from further analysis. Noisy channels were excluded (in four datasets, up to three electrodes were excluded). Infomax independent component (IC) analysis was applied to correct for residual artifacts (e.g., eye blinks, eye movements, or tonic muscle activity). On average, 3.8 ICs were discarded (range: 1–8 ICs). Data of rejected channels were interpolated using neighboring electrodes. On average 62.9 spatial/remembered trials (range: 33–96), 45.7 nonspatial/remembered trials (range: 23–74), 34.7 spatial/forgotten trials (range: 19–61), and 49.7 nonspatial/forgotten trials (range: 22–81) passed artifact corrections.

Data were filtered using wavelets with a 5 cycle length to obtain oscillatory power between 2 and 30 Hz. The resulting data were *z*-transformed to respective mean and SD of power across the time dimension (i.e., across all trials of each frequency band and channel). For each subject and condition (spatial-remembered, spatial-forgotten, nonspatial-remembered, nonspatial-forgotten), all trials were averaged and smoothed with Gaussian kernel (FWHM: 200 ms and 2 Hz) to attenuate interindividual differences and to control for the time–frequency resolution trade-off across frequencies.

Source analysis was performed using a linearly constrained minimal variance (LCMV) beamformer (Van Veen et al., 1997), calculating a spatial filter based on the whole length of all trials. For all subjects, a standard source model with a grid resolution of 12 mm based on the Montreal Neurological Institute (MNI) brain and standard electrode positions realigned to the MNI MRI was used. Theoretically, EEG and MEG are equally suitable for source reconstruction (Michel and Murray, 2012). EEG source reconstruction in contrast to MEG source reconstruction critically relies on correct source models. However, standard head models have been shown to provide a suitable resolution (Fuchs et al., 2002). The source time-course for each grid point was calculated and subjected to a wavelet analysis and z-transformed similar to electrode level data. For virtual electrode analysis, data across all grid voxels covering the region of interest (ROI) were averaged. Grid voxel data were interpolated to a 2 mm resolution single-subject MNI brain for plotting and to define the locations of clusters and peaks.

For statistical analysis of EEG data, power spectra for each subject were collapsed and averaged across all trials for each “cell” of the design matrix (spatial-remembered, spatial-forgotten, nonspatial-remembered, and nonspatial-forgotten). All of the following EEG analyses are based on these first-level averages (4 cells × 21 subjects). This prior averaging for each cell for each subject controls for possible trials biases in the analysis of main effects (e.g., more spatially remembered trials than nonspatially remembered trials, if all remembered trials, irrespective of condition, would be pooled for investigating memory effects).

As statistical tests, nonparametric cluster permutation tests were used as implemented in fieldtrip (Maris and Oostenveld, 2007). The cluster permutation test consists of the following two steps: first, clusters of coherent *t* values exceeding a certain threshold (here *p* < 0.05) along selected dimensions (time, frequency, electrodes/grid voxels) are detected in the data. Second, summed *t* values of these clusters are then compared to a null distribution of *t* sums of random clusters obtained by permuting condition labels across subjects. This procedure effectively controls for type I errors due to multiple testing. The clusters of *t* values subjected to permutation testing can be built across different dimensions: clustering can be performed on nonaveraged data across all dimensions (electrode, frequency, time) or a specific dimension when averaging over certain dimensions (i.e., averaging in the time–frequency window and then clustering across the electrode dimension).

Clustering was employed along different dimensions depending on the data. In a first step to openly identify the time–frequency window of theta effects, a three-dimensional clustering approach was used (time × frequency × electrodes) restricted around the frequency band of interest (FOI analysis, 1–10 Hz, encompassing the theta band ∼3–7 Hz). For identification of time–frequency windows showing significant differences between conditions across all lower frequencies (1–30 Hz) in a more explorative/liberal manner a sliding window cluster permutation test was used (for details, see Staudigl and Hanslmayr, 2013). Here, a cluster permutation test clustering across electrodes for every 300 ms × 1 Hz time–frequency is calculated sliding across all lower frequencies in 100 ms and 0.5 Hz steps. This approach yields a *p* value for each of these time–frequency bins (see Fig. 3B). Only effects consisting of coherently significant bins larger than 500 ms and 2 Hz (i.e., 3 time × 4 frequency bins) were subjected to further analysis. The average power of time–frequency windows identified in the theta-restricted cluster statistic and the sliding cluster statistic were subjected to an additional cluster permutation test clustering across electrodes in order to capture the topographies of effects and to check the spatial and temporal stability of effects. Analysis of whole-brain source-localized effects was also performed for the average activity in the time–frequency windows identified in scalp analysis, clustering now along the spatial dimension (i.e., source grid voxels). For analysis in virtual electrodes, we used two-dimensional clustering (time × frequency).

Task contrasts of interest were interaction effects and main effects of the 2 × 2 repeated-measurements design (i.e., power spectrum for condition × memory). In order to stay within the fieldtrip cluster statistic framework, these contrasts were calculated using the cluster permutated *t* contrasts. The interaction effect was tested contrasting the difference between remembered and forgotten trials between both encoding conditions. The main effect of memory was analyzed by contrasting the average of spatial-remembered and nonspatial-remembered averages with the average of spatial-forgotten and nonspatial-forgotten averages. The main effect of the encoding condition was respectively tested by contrasting means of the spatial condition with means of the nonspatial condition. This analysis scheme of *t* tests for testing interaction and main effects is equivalent to a 2 × 2 repeated-measures ANOVA.

### fMRI recording

Imaging was performed using a 3 tesla MR head scanner (Siemens Allegra). During fMRI scanning, 2475–2480 whole-brain images, consisting of 34 axial slices, were continuously acquired using an interleaved, standard T2*-weighted echoplanar imaging sequence [repetition time (TR): 2000 ms; echo time (TE): 30 ms; flip angle, 90°; 64 × 64 matrices; in-plane resolution, 3 × 3 mm; slice thickness, 3 mm]. High-resolution (1 mm isotropic voxel size) sagittal T1-weighted images were acquired after the functional scans, using a magnetization-prepared rapid gradient echo sequence (TR: 2250 ms; TE: 2.6 ms) to obtain a 3D structural scan.

### fMRI preprocessing and analysis

Image preprocessing and statistical analysis were performed using SPM8 (Wellcome Department of Cognitive Neurology, London. UK; www.fil.ion.ucl.ac.uk/spm; RRID: SCR_007037), running on MATLAB (version 2012b, MathWorks). After discarding the first two images of each session, time series were corrected for differences in slice acquisition time, spatially realigned to the first image of the session, and unwarped. The mean functional image was coregistered to the structural image. Global effects in the functional time series within each session and voxel were removed using linear detrending (Macey et al., 2004). All functional images were then normalized to MNI space (www.mni.mcgill.ca) using the normalization parameters determined from segmentation of the structural image. As a last step, images were smoothed with a Gaussian kernel of 8 mm (FWHM).

Scans of all four sessions were concatenated in first-level GLMs. Activity related to the subsequent memory and encoding task was modeled by event-related stick regressors for each condition (spatial-remembered, spatial-forgotten, nonspatial-remembered, nonspatial-forgotten) convolved with the canonical first-order hemodynamic response function. Further regressors of no interest were modeling the rest periods (30 s breaks between encoding–recall blocks), free-recall periods (separately for each encoding condition), and the distracter task. Session-specific regressors, linear drifts within each session, and movement parameters determined during realignment were also included in the model. Contrasts capturing encoding effects for the four conditions of interest were calculated in each single participant, and combined in a 2 (task) × 2 (subsequent memory) full-factorial random-effects model on a group level.

As a first step, an ROI analysis on MTL effects was performed using small volume correction on bilateral MTL as defined by the Wake Forest University WFU_PickAtlas (https://www.nitrc.org/projects/wfu_pickatlas/; RRID: SCR_007378; parahippocampal cortices plus hippocampi using aal atlas, RRID: SCR_003550, the same ROI definition as used for virtual electrodes ROI analysis). Small volume-corrected effects are reported using *p* < 0.001, cluster size of >10 voxels, cluster *p* level, familywise error (FWE) corrected to <0.05. An additional whole-brain analysis was carried out using a threshold of *p* < 0.001 uncorrected, cluster *p* level, familywise error corrected <0.05. MarsBaR (http://marsbar.sourceforge.net; RRID: SCR_009605) was used to extract parameter estimates of the left MTL ROI (see Fig. 7). Results are plotted on the mean normalized structural scan of all subjects.

## Results

### Behavioral performance

**Figure 2.**
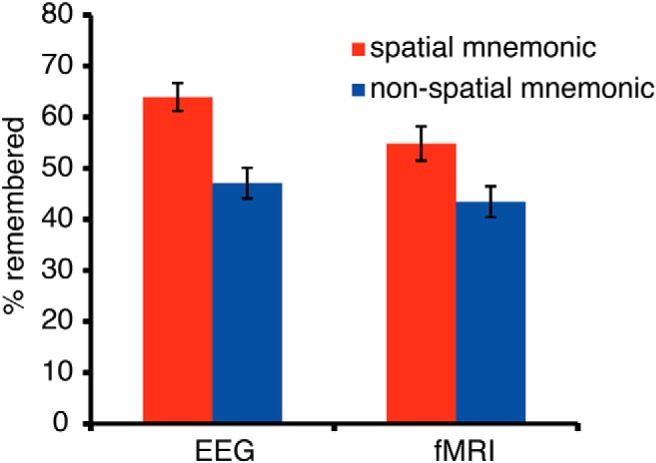
Memory performance: the percentage of recalled words in the spatial and nonspatial encoding condition during the EEG experiment and fMRI experiment. In both datasets, memory performance was higher using the spatial method of loci mnemonic. Error bars show the SEM.

Memory performance in EEG and fMRI was reasonably high, in line with the efficiency of mnemonic strategies (Roediger, 1980). Memory performance (Fig. 2) in both datasets was significantly higher during the spatial encoding than during the nonspatial encoding task (fMRI: *t*_(22)_ = 6.26, *p* < 0.0001; EEG: *t*_(20)_ = 10.23, *p* < 0.0001). This was a very robust effect, which was visible in almost every single subject: 19 of 21 EEG participants and 20 of the 23 fMRI participants showed higher recall rates using the spatial mnemonic, demonstrating the power of the method of loci as a mnemonic (Roediger, 1980).

### EEG scalp level

**Figure 3.**
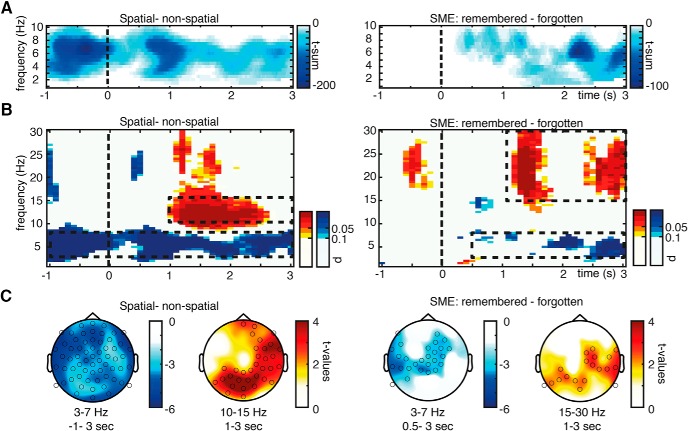
EEG sensor level results. ***A***, A cluster permutation statistic restricted to the theta frequency range revealed ongoing decreases in theta oscillatory power for spatial mnemonic processing in contrast with nonspatial processing, and item-related theta power decreases correlating with successful memory formation. The time–frequency plots show the *t*-sum values across electrodes of the significant clusters at every time–frequency bin to visualize the extent of the three-dimensional clusters. ***B***, Additional increases in alpha/beta power during spatial encoding and memory formation were evident after word presentation. Time–frequency plots here show *p* values of a sliding cluster statistic (i.e., separately calculated cluster permutation tests of each time–frequency bin). ***C***, Topographies of theta and alpha/beta power effects of a cluster statistic for the average power for time–frequency windows highlighted in ***B*** (dashed boxes) are plotted below, circles highlight electrodes belonging to significant clusters. Warm colors indicate increases in power for spatial processing and successfully encoded items, cold colors indicate decreases in power for spatial processing and successfully encoded items in contrast to nonspatial processing and subsequently forgotten items, respectively.

The first step of analysis focused on theta frequency range as this frequency band has been previously linked to spatial processing and memory formation. Therefore, we contrasted theta power during the spatial and nonspatial mnemonic and for remembered and forgotten trials (SME; Fig. 3*A*). A robust theta power decrease for the spatial mnemonic contrasted to the nonspatial encoding strategy was found spanning the whole trial epoch (−1 to 3 s, *p*_corr_ = 0.002; Fig. 3*A*). This theta power decrease was ongoing throughout the trial and does not appear to be triggered by the onset of the to-be-encoded words. Decreases in theta power were also related to successful memory formation as revealed by negative SMEs. A significant cluster of memory-related theta decreases was evident from stimulus onset lasting until the end of the trial (0.5–3 s; *p*_corr_ = 0.014; Fig. 3A). The extent of the clusters is shown in Figure 3A (left for spatial vs nonspatial; right for SMEs). This pattern of results shows that spatial mnemonic processing as well as successful memory formation is reflected in theta power decreases.

An additional sliding cluster permutation statistic was performed on all lower-frequency bands (1–30 Hz; Fig. 3*B*) to investigate effects outside of the frequency of interest in an exploratory manner. Significant effects were evident in the alpha/beta range (10–30 Hz; Fig. 3*B*). Here, a stronger power increase was evident after stimulus presentation (1–3 s) for the spatial mnemonic relative to the nonspatial strategy (10–15 Hz). Similarly a positive alpha/beta SME indicating higher alpha/beta power during successful memory formation was evident (15–30 Hz, 1–3 s; Fig. 3*B*).

For a first identification of potential sources of EEG effects, an additional cluster statistic across electrodes was carried on the average power in the theta and alpha/beta time–frequency windows identified in the prior analyses. Topographies of the spatial clusters obtained are shown in Figure 3*C*. The decrease in theta power related to the spatial mnemonic showed a widespread topography with the strongest effects over lateral electrodes (*p*_corr_ = 0.002). Theta power decreases related to memory encoding were evident in left temporal lateral and right frontal regions (*p*_corr_ = 0.002). Stronger increases in alpha/beta power during the spatial mnemonic were found over parietal electrodes (*p*_corr_ = 0.004). Similar to the spatial processing-related alpha/beta effects, memory-related alpha/beta power increases showed a posterior and right lateralized topography (*p*_corr_ = 0.042).

To investigate potential differences of encoding effects between the spatial and nonspatial mnemonic strategies, interaction effects were calculated by contrasting SMEs of both conditions. No significant clusters were found in a cluster analysis in the theta frequency range (1–10 Hz, similar analysis as for Fig. 3*A*). Also, an additional sliding cluster statistic including all lower frequencies (2–30 Hz similar analysis as for Fig. 3*B*) revealed no coherent effects exceeding a size of eight time–frequency bins.

This lack of interaction effects shows that the difference in theta power between encoding conditions is not dependent on memory performance. This was additionally tested using a cluster analysis in the theta frequency range (1–10 Hz) contrasting the spatial and nonspatial condition restricted to remembered trials. This contrast of spatial-remembered and nonspatial-remembered conditions showed a very similar pattern as the condition difference spatial versus nonspatial: strong theta decreases throughout the trial epoch (*p* = 0.002; compare Fig. 3*A*). This result can be inferred from the pattern of main and interaction effects and therefore is not additionally plotted.

Furthermore, the lack of interaction effects indicated no differences in SMEs for spatial and nonspatial processing. This suggests that successful encoding in both mnemonic strategies similarly relies on theta power decreases and that indeed theta decreases are related to associative memory encoding irrespective of the spatial nature of the task.

### fMRI results

**Figure 4.**
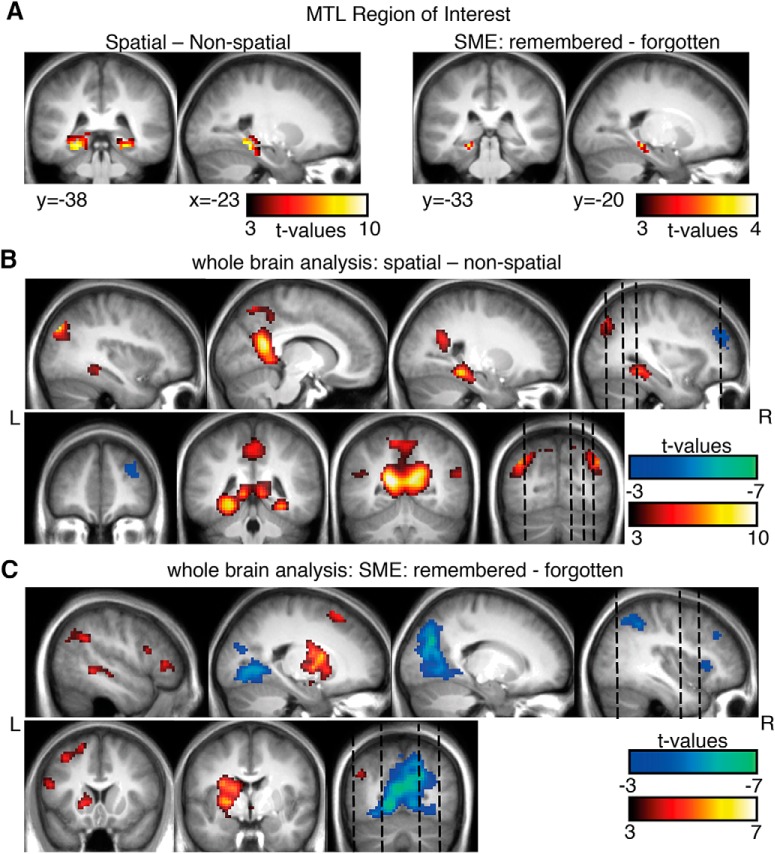
fMRI results for spatial vs nonspatial contrasts and memory effects. ***A***, A region of interest analysis was performed for MTL regions, revealing increases in activity for the spatial mnemonic and successful memory formation (*p* < 0.001, cluster size >10). ***B***, ***C***, An exploratory whole-brain analysis revealed additional effects in typical spatial cortical networks (i.e. retrosplenial cortex, bilateral MTL, ***B***) and memory related regions (i.e., left inferior frontal gyrus, ***C***; *p* < 0.001, all *p* < 0.05 FWE cluster level). Warm colors indicate higher BOLD signals for spatial processing and later remembered items; cold colors indicate higher BOLD signals for nonspatial processing and subsequently forgotten items.

As a first step, we investigated MTL BOLD changes. To this end, an ROI analysis on anatomically predefined MTL regions was performed (Fig. 4*A*, left; Table 1). BOLD activity increased significantly in bilateral parahippocampal gyrus (PHG) during the spatial mnemonic in contrast to the nonspatial task. The significant clusters were spanning parahippocampal regions as well as hippocampus (left MTL: 68 voxels in PHG; 47 voxels in hippocampus; right MTL: 74 voxels in PHG; 33 voxels in hippocampus). Positive SMEs were found in the left MTL (Fig. 4*A*, right; Table 1) restricted to the parahippocampal gyrus at the current statistical threshold (left MTL: PHG, 15 voxels). In line with our hypotheses, these results suggest that the spatial mnemonic and successful associative memory formation both rely on increased hemodynamic MTL activity.

**Table 1:** Locations of peak activation revealed in MTL ROI analysis

	hs	BA	Size	MNI coordinates			*t*
				*x*	*y*	*z*	
**Spatial > nonspatial**							
Parahippocampal gyrus	L	36	127	−24	−37	−11	9.15
	L			−24	−31	−20	5.41
Parahippocampal gyrus	R	36	112	27	−37	−11	8.32
**positive SME: remembered > forgotten**							
Parahippocampal gyrus	L	35	16	−21	−34	−11	3.79
	L	35		−24	−28	−20	3.38

BA, Brodmann area; hs, hemisphere; L, Left; R, right.

**Table 2:** Locations of peak activation revealed in the whole-brain analysis

	hs	BA	Size	MNI coordinates			*t*
				*x*	*y*	*z*	
**Spatial > nonspatial**							
Posterior cingulate	L	30	1999	−15	−58	16	12.64
Parahippocampal gyrus	L	36		−24	−40	−11	9.67
Posterior cingulate	R	30		12	−52	16	9.56
Superior occipital gyrus	L	19	186	−36	−76	34	8.78
Superior temporal gyrus	L	22		−45	−55	22	3.91
Middle temporal gyrus	L	39		−54	−67	22	3.59
Parahippocampal gyrus	R	36	206	27	−37	−14	8.54
Middle temporal gyrus	R	39	215	42	−73	34	6.89
Superior temporal gyrus	R	39		57	−58	22	5.19
Middle temporal gyrus	R	39		48	−67	25	4.92
**Nonspatial > spatial**							
Middle frontal gyrus	R	9	102	30	47	28	4.23
	R	9		45	29	22	3.76
**Positive SME: remembered > forgotten**							
Insula	L	13	608	−30	−7	19	5.77
Caudate body	L			21	5	13	5.68
Putamen	L			−24	−4	1	5.58
Middle frontal gyrus	L	6	141	−24	23	52	4.83
	L	6		−28	17	58	4.61
	L	6		−36	17	46	4.49
Middle temporal gyrus	L	22	86	−51	−43	−2	4.56
	L	21		−60	−19	−5	4.01
Superior temporal gyrus	L	22		−48	−25	−5	3.77
Middle temporal gyrus	L	39	114	−42	−73	31	4.44
Superior temporal gyrus	L	39		−48	−52	31	4.41
Inferior frontal gyrus	L	44	135	−51	14	19	4.37
	L	13		−45	32	1	4.36
	L			−45	47	−5	3.93
**Negative SME: forgotten > remembered**							
Lingual gyrus	L	18	2237	−12	−70	−2	7.25
Posterior cingulate	L	30		−3	−70	16	6.98
Cuneus	R	18		9	−73	25	6.67
Inferior parietal lobule	R	40	355	42	−46	46	4.94
Superior parietal lobule	R	7		33	−52	49	4.41
Supramarginal gyrus	R	40		57	−43	37	4.41
Middle frontal gyrus	R	10	96	30	62	13	4.36
	R	8		39	35	34	4.08
Superior frontal gyrus	R	9		27	53	34	3.67
Insula	R	13	68	36	26	1	4.36

L, Left; R, right.

**Table 3:** Statistical table

Figure/section	Description/data structure	Test	Statistical value	*p* value
**Behavioral analysis**				
Methods: task design	Number of trials remembered out of sequence in EEG: average of out of sequence trials spatial vs nonspatial	Paired *t* test	*t*_(20)_ = 0.4	0.68
	Number of trials remembered out of sequence in fMRI average of out of sequence trials spatial vs nonspatial	Paired *t* test	*t*_(22)_ = −0.168	0. 87
Results: Behavioral Performance and Figure 2	Memory performance EEG: relative number of remembered items spatial vs nonspatial	Paired *t* test	*t*_(20)_ = 10.23	0.0001
	Memory performance fMRI: relative number of remembered items spatial vs nonspatial	Paired *t* test	*t*_(22)_ = 6.26	0.0001
**EEG analysis**				
Results: EEG scalp level and Figure 3A	FOI analysis theta band: SME: forgotten vs remembered trials, 1–10 Hz, −1 to 3 s	3D cluster permutation statistic	One sig. neg. cluster *t*_sum_ = −55,635	*p*_corr_ = 0.03
	FOI analysis Theta band: condition difference, spatial vs Nonspatial, 1–10 Hz, −1 to 3 s	3D cluster permutation statistic	One sig. neg. cluster *t*_sum_ = −287,990	*p*_corr_ = 0.002
	FOI analysis Theta band: Interaction SME spatial vs SME nonspatial, 1–10 Hz, −1 to 3 s	3D cluster permutation statistic	no sig. cluster	Min. *p*_corr_ = 0.68
	FOI analysis theta band: spatial remembered vs nonspatial remembered, 1–10 Hz, −1 to 3 s	3D cluster permutation statistic	One sig. neg. cluster T_sum_ = −158380	*p*_corr_ = 0.002
Results: EEG scalp level and Figure 3B	SME: forgotten vs remembered trials, 1–30 Hz, −1 to 3 s	Sliding cluster permutation statistic	Coherent sig. bins in alpha/beta band	
	Condition difference: spatial vs nonspatial, 1–30 Hz, −1 to 3 s	Sliding cluster permutation statistic	Coherent sig. bins in alpha/beta band	
	Interaction: SME spatial vs SME nonspatial, 1–30 Hz, −1 to 3 s	Sliding cluster permutation statistic	no coherent sig. bins (min. 3 time × 4 frequency bins)	
Results: EEG scalp level and Figure 3C	Topoplot: SME theta, 3–7 Hz, 0.5–3 s	1D cluster permutation statistic	One sig. neg. cluster *t*_sum_ = −75.85	*p*_corr_ = 0.004
	Topoplot: SME alpha/beta, 15–30 Hz, 1–3 s	1D cluster permutation statistic	Two sig. pos. clusters *t*_sum_ = 29.60 and 19.34	*p*_corr_ = 0.012, *p*_corr_ = 0.042
	Topoplot: condition difference theta 3–7 Hz, −1 to 3 s	1D cluster permutation statistic	One sig. neg. cluster *t*_sum_ = −239.30	*p*_corr_ = 0.002
	Topoplot: condition difference alpha/beta, 10–15 Hz, 1–3 s	1D cluster permutation statistic	One sig. pos. cluster *t*_sum_ = 104.43	*p*_corr_ = 0.012
Results: EEG source analysis and Figure 5	Virtual electrode statistic: SME theta in right MTL, 2–10 Hz, −1 to 3 s	1D cluster permutation statistic	No sig. cluster	Min. *p*_corr_ = 0.20
	Virtual electrode statistic: SME theta in left MTL, 2–10 Hz, −1 to 3 s	1D cluster permutation statistic	One sig. neg. cluster *t*_sum_ = −246.33	*p*_corr_ = 0.014
	Virtual electrode statistic: condition difference in right MTL, 2–10 Hz, −1 to 3 s	1D cluster permutation statistic	Two sig. neg. clusters T_sum_ = −588.36 and −562.18	*p*_corr_ = 0.006, *p*_corr_ = 0.006
	Virtual electrode statistic: condition difference in left MTL, 2–10 Hz, −1 to 3 s	1D cluster permutation statistic	Two sig. neg. clusters *t*_sum_ = −682.99 and −516.30	*p*_corr_ = 0.002, *p*_corr_ = 0.004
Results: EEG source analysis and Figure 6				
	Source statistic: SME theta	1D cluster permutation statistic	One sig. neg. cluster *t*_sum_ = −3140.3	*p*_corr_ = 0.003
	Sourcestatistic: SME alpha/beta	1D cluster permutation statistic	One sig. pos. cluster *t*_sum_ = 433.72	*p*_corr_ = 0.054
	Sourcestatistic: condition difference alpha/beta	1D cluster permutation statistic	One sig. pos. cluster *t*_sum_ = 2692.8	*p*_corr_ = 0.0034
EEG and fMRI ROI analysis				
Results: EEG source analysis and Figure 7	SME: forgotten vs remembered, average theta power MTL 3–7 Hz, −1 to 3 s	2 × 2 ANOVA, main effect	*F*_(1,20)_ = 10.08	0.005
	condition difference: spatial vs nonspatial, average theta power lMTL 3–7 Hz, −1 to 3 s	2 × 2 ANOVA, main effect	*F*_(1,20)_ = 43.87	<0.0001
	Interaction: SME spatial vs SME nonspatial, average theta power lMTL 3–7 Hz, −1 to 3 s	2 × 2 ANOVA, interaction effect	*F*_(1,20)_ = 0.001	0.97
Results: EEG source analysis and Figure 7	SME: forgotten vs remembered, average beta weights MTL	2 × 2 ANOVA, main effect	*F*_(1,22)_ = 10.20	0.004
	condition difference: spatial vs nonspatial, average beta weights MTL	2 × 2 ANOVA, main effect	*F*_(1,22)_ = 16.23	0.001
	Interaction: SME spatial vs SME nonspatial average beta weights MTL	2 × 2 ANOVA, interaction effect	*F*_(1,22)_ = 2.84	0.13

Min., Minimum; neg., negative; pos., positive; sig., significant.

An additional exploratory whole-brain analysis (Fig. 4*B*,*C*; Tables 2) was performed to investigate which other regions are involved during mnemonic encoding. Regions typically associated with spatial processing (Burgess et al., 2002; Epstein, 2008) showed BOLD signal increases during spatial compared with nonspatial processing (Fig. 4*B*): bilateral retrosplenial cortex (posterior cingulate cortex, BA 30), and lateral temporal areas of the angular gyrus (BA 39). In contrast, the right middle frontal gyrus (Fig. 4*B*) showed relative increases in activity during the nonspatial mnemonic, potentially related to enhanced control processes (Schott et al., 2005). Positive subsequent memory effects (Fig. 4*C*) were found in the left hemisphere in inferior frontal gyrus, an area typically involved in memory encoding of verbal material (Kim, 2011). Additional memory-related activity was evident in striatal areas (putamen, caudate body) and the left superior and middle temporal gyrus. Relative decreases in BOLD signals during remembered words contrasted with forgotten words (i.e., negative SMEs) were evident in bilateral occipital areas (cuneus, lingual gyrus, areas posterior to the more retrosplenial centered spatial vs nonspatial effects) and right lateralized parietal and frontal regions (Fig. 4*C*).

To elucidate whether memory encoding-related activity differs between the spatial and nonspatial mnemonic strategies, interaction effects were calculated. No significant differences in memory encoding between encoding strategies (i.e., no interaction effects between condition and memory) were found in the ROI analysis or in the whole-brain analysis (*p* < 0.001, cluster size >10, uncorrected). Spatial and nonspatial encoding therefore seem to rely on BOLD activity in similar brain regions. This absence of differences in encoding-related activity in fMRI results closely matches the absence of differences in EEG findings. This finding indicates that associative memory formation relies on MTL BOLD increases and theta power decreases, irrespective of whether associations were formed using a spatial or nonspatial encoding strategy.

### EEG source analysis

**Figure 5.**
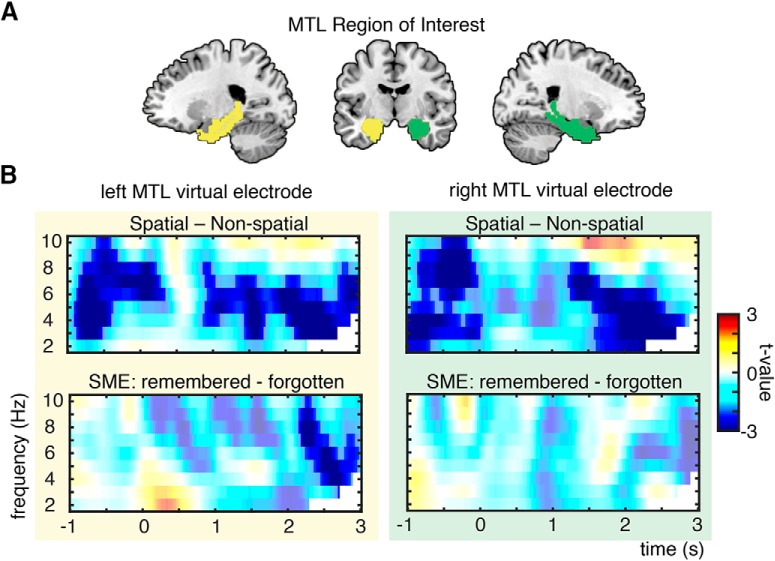
Theta power changes in MTL. ***A***, MTL region of interest consisting of parahippocampal gyrus and hippocampus, highlighted here in green (right MTL) and yellow (left MTL). Virtual electrodes were placed in the same ROIs as in fMRI ROI analysis (Figs. 4A, 7). ***B***, Theta power effects of virtual electrodes in left and right MTL: theta power decreases were found bilaterally for spatial vs nonspatial processing and left lateralized for successful memory formation. Nonsignificant time–frequency bins are whitened.

Based on our hypotheses and backed up by the fMRI results, we placed virtual electrodes in bilateral MTL regions to investigate the effects of theta power on source level. Oscillatory source effects were estimated by means of a source localization analysis using LCMV beamforming (Van Veen et al., 1997), and virtual electrode placement was based on the region of interest of fMRI analysis in left and right MTL (Fig. 5*A*). In bilateral MTL, theta power showed stronger decreases prestimulus and poststimulus during spatial mnemonic processing than during the nonspatial strategy (left MTL, two clusters: *p*_corr_ = 0.002 and *p*_corr_ = 0.004; right MTL, two clusters: *p*_corr_ = 0.006 and *p*_corr_ = 0.006; Fig. 5*B*). SMEs in the theta band were evident only in the left MTL: theta power decreases were significant poststimulus (*p*_corr_ = 0.022; Fig. 5*B*), but not in the right MTL (all clusters, *p*_corr_ > 0.2). This pattern of bilateral MTL effects during spatial processing and left lateralized MTL effects during memory formation parallels the fMRI MTL results (Fig. 4*A*).

**Figure 6.**
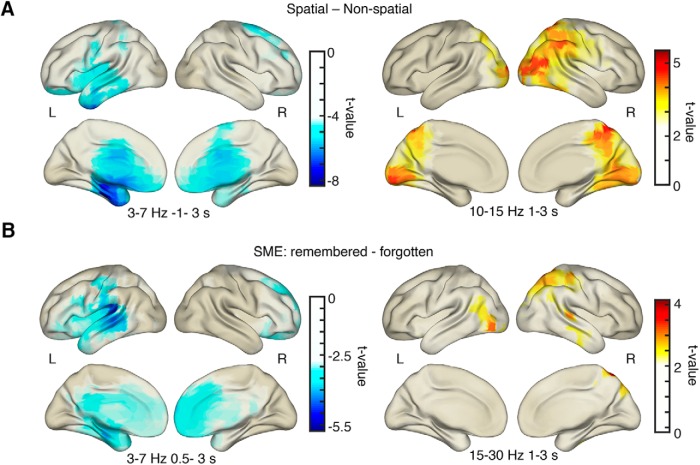
EEG source localization results. ***A***, Decreases in theta power for spatial processing were strongest in anterior MTL areas, Alpha/beta power increases were strongest in occipital–parietal–temporal areas for spatial vs nonspatial processing. ***B***, Theta power decreases during successful memory formation were strongest in left temporal areas. Increases in alpha/beta power during memory formation were found in occipital–parietal areas. All plots are thresholded at half-maximum *t* value.

To illustrate the whole-brain distribution of power effects, peaks of source-localized task and memory-related oscillatory activity are shown in Figure 6. Theta power decreases for spatial mnemonic processing compared with nonspatial processing were strongest in the left anterior temporal lobe (*p*_corr_ = 0.001; *t*_peak_ = −8.34; peak MNI coordinates: *x* = −31, *y* = −19, *z* = −31; fusiform gyrus; Fig. 6*A*). Memory formation-related theta power decreases were strongest in left lateral temporal lobe areas (*p*_corr_ = 0.003; *t*_peak_ = −5.76; peak MNI coordinates: *x* = −76, *y* = −18, *z* = 5; middle temporal gyrus; Fig. 6*A*). Note the overlap of theta power decreases and BOLD activity increases in MTL and temporal regions (compare Figs. 6A, 4).

Alpha/beta power increases for spatial mnemonic processing and positive SMEs were found in occipito-parietal areas and right lateralized regions (spatial-nonspatial: *p*_corr_ = 0.003; *t*_peak_ = 5.52; peak MNI coordinates: *x* = 18, *y* = −55, *z* = 77; superior parietal gyrus; SME: *p*_corr_ = 0.054; *t*_peak_ = 4.31; peak MNI coordinates: *x* = 18, *y* = −66, *z* = 65; superior parietal gyrus; Fig. 6*B*). Note that the alpha/beta memory effect is only marginally significant on the source level. Similar to theta decreases and BOLD increases, alpha/beta power and BOLD activity seem to be negatively related: increases in alpha/beta power overlap with areas where BOLD decreases during successful memory formation were evident (compare Figs. 6B, 4C).

**Figure 7.**
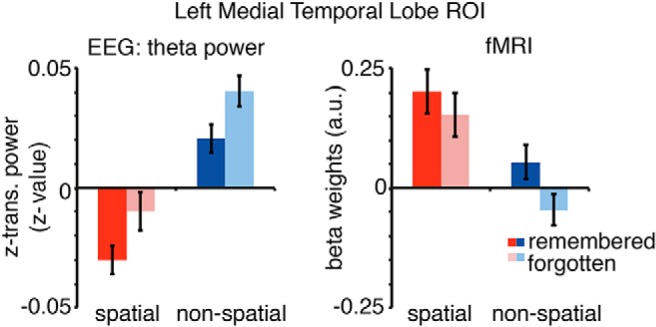
Theta EEG power and fMRI beta weights of the left MTL ROI. Theta power (3–7 Hz, −1 to 3 s) and beta weights were averaged for each condition for all voxels included in the anatomically defined left MTL ROI. Theta power decreases show the reversed pattern of BOLD increases in left MTL regions. Error bars show the SEM.

To further illustrate the relationship between the MTL BOLD signals and theta power, average EEG theta power and the corresponding fMRI beta estimates derived from the left MTL ROI as also used in fMRI ROI analysis are plotted in Figure 7. Using this anatomically defined ROI, a similar pattern of effects is evident in both modalities: SMEs in both encoding conditions do not differ (interaction effect fMRI: *F*_(1,22)_ = 2.481, *p* = 0.13, EEG: *F*_(1,20)_ = 0.001, *p* = 0.973). However, there is a significant difference between encoding conditions (main effect spatial vs nonspatial: fMRI: *F*_(1,22)_ = 16.24, *p* = 0.001; EEG: *F*_(1,20)_ = 43.67, *p* = 0.0001) and significant SME (main effect memory: fMRI: *F*_(1,22)_ = 10.20, *p* = 0.004; EEG: *F*_(1,20)_ = 10.08, *p* = 0.005). Decreases in theta power mirror fMRI effects such that contrast showing decreases in theta power are associated with fMRI contrast revealing MTL BOLD signal increases.

All statistical test carried out on the data are listed in Table 3.

## Discussion

Brain oscillations in the theta range and hemodynamic activity in the medial temporal lobe are considered to be core neural correlates of successful memory formation in electrophysiological and functional imaging studies, respectively. We here addressed the question of how theta power and MTL BOLD signals are functionally linked. To this end, we measured EEG and fMRI during two associative encoding strategies: the spatial method of loci and the nonspatial pegword method. In line with previous work, the spatial mnemonic indeed showed stronger increases in BOLD signal in bilateral parahippocampal gyrus and hippocampus relative to the nonspatial condition. Strikingly, these MTL BOLD signal increases were paralleled by MTL theta power decreases. Successful encoding during both mnemonics was predicted by equally pronounced left MTL BOLD signal increases restricted to the parahippocampal gyrus, which were paralleled by theta power decreases in left lateral and medial temporal regions. Together, these findings suggest a negative relationship among theta power, memory formation, spatial processing, and MTL BOLD signals.

The method of loci recruits almost all cognitive processes that have been linked to MTL functions (i.e., scene construction, spatial processing, imagery, memory encoding and retrieval, and building new associative links; Bird and Burgess, 2008), which renders the method of loci ideal for studying MTL theta oscillations during memory encoding. Theta power decreases were source localized in areas overlapping with fMRI findings (Table 1). Peaks of source-localized theta power decreases were found in anterior MTL and in left lateral temporal areas. Analysis of theta power changes in virtual electrodes placed in the right and left MTL ROIs closely followed the fMRI results (Figs. 4, 5). Spatial processing in contrast to nonspatial processing led to bilateral MTL theta decreases and BOLD increases, and subsequent memory effects in theta power and BOLD activity were evident in the left MTL. These parallels indicate that fMRI BOLD and EEG power can be linked via source localization techniques (Singh, 2012).

FMRI effects during spatial encoding closely follow previously reported effects relating to the usage of mnemonic strategies (Maguire et al., 2003) and spatial processing in general. The spatial mnemonic exhibited increases in BOLD signal in bilateral MTL and retrosplenial cortex, regions that are typically involved in imagining scenes and retrieving familiar landmarks (Burgess et al., 2002; Epstein, 2008). The method of loci task requires constructing spatial scenes to link to-be-learned items to familiar landmarks. Constructing such complex visual scenes crucially involves MTL structures (Addis et al., 2007; Hassabis and Maguire, 2007) with the retrosplenial cortex as a buffer of such imagined constructions (Bird and Burgess, 2008; Vann et al., 2009). The greater involvement of these brain structures in spatial processing, which additionally serves a central function in human memory (Hassabis and Maguire, 2007), might be partly responsible for the efficiency of the method of loci in general (Roediger, 1980) and for better memory performance during spatial processing.

Interestingly, subsequent memory effects in both mnemonics did not significantly differ depending on the spatial or nonspatial nature of the mnemonic, either in EEG or in fMRI. Memory formation during both tasks was related to activity in left MTL, which is consistent with the notion that the human MTL is involved in building item–cue associations (Ranganath, 2010; Buzsáki and Moser, 2013), regardless of the nature of these cues (i.e., spatial or nonspatial). Successful formation of associations in imagined navigational space and in semantic space thus seems to rely on similar mechanisms. This pattern of similar encoding effects for both encoding strategies supports the idea that humans use similar MTL mechanisms in spatial processing and in episodic memory encoding (Buzsáki and Moser, 2013; Ekstrom, 2014; Buffalo, 2015). Furthermore, the findings indicate that an encoding strategy like the method of loci elicits an “encoding-friendly” state (i.e., MTL BOLD increases and theta power decreases), which likely enhances memory performance.

The effects in the alpha/beta frequency, although not part of the initial frequency band of interest nicely match prior reported effects. The finding of posterior alpha/beta power increases paralleling BOLD signal decreases is well in line with several studies showing a negative relationship between low-frequency power (<30 Hz) and BOLD signal (Mukamel et al., 2005; Hanslmayr et al., 2011; Scheeringa et al., 2011; Hermes et al., 2014; Zumer et al., 2014). These findings are compatible with those of previous studies showing that occipital alpha/beta power increases predict long-term memory formation during internal processing of to-be-memorized material (Khader et al., 2010; Meeuwissen et al., 2011). During the use of the mnemonic strategies, subjects have to maintain and manipulate the to-be-encoded item and the spatial or nonspatial cue. During the formation of these spatial or nonspatial internal scenes, attention might be actively switched inward by inhibiting the occipital cortex to prevent task-interfering visual input (Klimesch et al., 2007; Jensen and Mazaheri, 2010). Such an inhibition of posterior processing regions might be reflected by decreases in BOLD activity accompanied by posterior alpha/beta power increases, which ultimately benefits memory (Scheeringa et al., 2009). An open question is how these reported increases in alpha/beta power relate to the commonly found alpha/beta decreases during memory formation (Hanslmayr et al., 2011). A possible explanation is that posterior increases in alpha power, the highest amplitude signal of the human EEG, cancel out frontal, memory encoding-related power decreases. This explanation, although hypothetical, fits with the posterior-centered topography of alpha/beta effects (Fig. 3*C*) and with the pattern of weak early decreases in alpha/beta power (Fig. 3*B*) prior to posterior power increases.

Studies on oscillatory correlates of memory often implicitly assume a positive relationship between theta power increases and reported MTL BOLD increases (Summerfield and Mangels, 2005; Osipova et al., 2006; Hanslmayr et al., 2011; Staudigl and Hanslmayr, 2013; Backus et al., 2016), which is notable given that several MEG/EEG and intracranial EEG studies indeed show theta power decreases in the MTL during memory formation (Sederberg et al., 2007; Guderian et al., 2009; Burke et al., 2013; Long et al., 2014; Greenberg et al., 2015; Crespo-García et al., 2016; Griffiths et al., 2016).

Of note, and in contrast with the current results, a prior simultaneous EEG-fMRI study reported theta power increases alongside BOLD signal increases in the right MTL (Hanslmayr et al., 2011). This study, however, does differ from the present study in several important aspects. In contrast to the present paradigm, this prior study used a task and recording procedure that was not specifically tailored toward the MTL. Importantly, despite reported BOLD correlations with power in the beta frequency band, the reported theta power increase did not show any correlations with BOLD. These problems are highlighted in this previous study, and it is proposed that the relationship between MTL BOLD signal and theta power should be investigated by future studies using more MTL-dependent tasks. Furthermore, the increase in theta power was found in data recorded simultaneously with the fMRI recording. EEG data, especially in the lower-frequency bands (below ∼10 Hz) is highly affected by MR-related artifacts (Debener et al., 2008; Fellner et al., 2016). Consequently, considering the present results and recent studies on spatial memory encoding (Crespo-García et al., 2016; Griffiths et al., 2016), decreases in theta power during memory formation seem to be the more stable finding, contradicting the finding of this prior study.

On a related note, studies investigating memory encoding investigate a wide range of different paradigms using different encoding and testing conditions and different materials, which can affect the direction of theta power SMEs (for a detailed discussion, see Hanslmayr and Staudigl, 2014). Interestingly, a recent study (Crespo-García et al., 2016) also investigating an MTL-related spatial-associative encoding task in MEG and intracranial recordings reported similar theta decreases correlating with memory formation. The present results cannot elucidate why prior studies found mixed results regarding theta power changes and memory encoding. However, the findings demonstrate a co-occurrence of MTL activity increases and theta power decreases and demonstrate that a task tailored toward maximally driving MTL activity does not elicit theta power increases, but instead elicits a decrease in theta power.

In line with these studies, and also with studies combining fMRI and electrophysiological measures (Mukamel et al., 2005; Niessing et al., 2005; Conner et al., 2011; Khursheed et al., 2011; Magri et al., 2012), the results suggest that the negative relationship between low-frequency power and BOLD power also extends to theta oscillations in the MTL during memory encoding (Lisman and Jensen, 2013). The resemblance of low-frequency power decreases during memory encoding in intracranial EEG (iEEG) to positive SMEs in fMRI has been noted before (Burke et al., 2013), albeit no study before has shown this overlap in scalp EEG. Recording EEG and fMRI in the same paradigm, the present results therefore support a functional link between theta power decreases and MTL BOLD increases, showing that these neural processes do not only occur in the same regions, but also covary with the same task conditions.

A central question remaining is how can we physiologically interpret theta power decreases paralleled by increases in BOLD signal? Traditionally, theta power increases have been hypothesized to reflect hippocampal–cortical feedback loops and increased neural cortico–hippocampal communication (Klimesch, 1996; Nyhus and Curran, 2010; Backus et al., 2016). Whether power increases are a suitable indicator of enhanced long-range neural communication, however, is questionable. For instance, intracranial recordings and MEG recordings during memory formation reported decreases in low-frequency theta power alongside increases in long-range phase synchrony (Burke et al., 2013; Crespo-García et al., 2016). A similar relationship of local power decreases and global synchrony increases has been shown in other experiments (Popov et al., 2013; Weisz et al., 2014). Furthermore, theta oscillations, which are dominant in the MTL and retrosplenial cortex during rest and movement (Ekstrom et al., 2009; Foster and Parvizi, 2012; Watrous et al., 2013b), might actually desynchronize in power during active tasks (Halgren et al., 1978; Mitchell et al., 2009), in order to flexibly form fine-grained cortical networks connecting cortical regions to specific MTL subregions (Watrous et al., 2013a; Maris et al., 2016). Since the EEG records a spatially smoothed attenuated sum of local field potentials (LFPs; Buzsáki et al., 2012), fine-grained long-range synchronizations of specific individual cortical–MTL networks might actually appear as power decreases in scalp EEG and iEEG recordings.

A direct mapping of EEG power effects onto BOLD signals is complicated. Both modalities correlate with LFPs (Logothetis et al., 2001; Buzsáki et al., 2012) but do not necessarily reflect the same neural processes (Ekstrom, 2010). Adding to the differences of physiological correlates between EEG and fMRI, both modalities differ substantially in their temporal and spatial resolution. Considering these constraints, our EEG localization and fMRI results still exhibited a relatively good fit despite the low spatial resolution of EEG (Fuchs et al., 2002) and the low temporal resolution of fMRI. The widespread topography of scalp-recorded EEG theta effects with temporal and frontal peaks is similar to topographies in prior studies reporting MTL sources of theta power effects (Staudigl and Hanslmayr, 2013; Backus et al., 2016) and also fits with the assumption that deep sources are reflected in more widespread sensor-level effects (Dalal et al., 2013). Our source localization results are also corroborated by studies showing MTL theta effects similarly in sensor-level source reconstructions and intracranial recordings directly in the MTL (Dalal et al., 2013; Crespo-García et al., 2016).

A limitation of the present study is that an analysis of simultaneously recorded theta oscillations during fMRI was not feasible due to strong artifacts induced by the MR scanning environment (Fellner et al., 2016). Therefore, EEG and fMRI were measured separately in independent subject samples, preventing the calculation of direct EEG–fMRI correlations. Nevertheless, the same paradigm was used in both datasets, and the same behavioral pattern of results was found across the two datasets. Theta power changes and MTL BOLD changes were evident in the same task contrasts and were overlapping spatially. Therefore, we can assume that, on average, the same cognitive processes were driving the EEG and fMRI effects in the two datasets (Mukamel et al., 2005; Singh, 2012).

### Conclusion

In summary, MTL and theta effects were more pronounced during the method of loci mnemonic, which indicates that navigating cognitive space might be a particularly efficient encoding strategy by maximally driving the neural processes related to spatial processing and episodic memory. The presented results show that decreases in theta oscillatory power in the MTL—similar to other cortical low-frequency oscillations (i.e., alpha/beta)—co-occur with neural activity as reflected in the BOLD signal. MTL activity in memory tasks therefore seem to map onto decreases in EEG theta power and not, as is often suggested, to increases in theta power.

## Synthesis

Reviewing Editor: Lila Davachi, New York University Decisions are customarily a result of the Reviewing Editor and the peer reviewers coming together and discussing their recommendations until a consensus is reached. When revisions are invited, a fact-based synthesis statement explaining their decision and outlining what is needed to prepare a revision will be listed below. The following reviewer(s) agreed to reveal their identity: Aidan Horner.

In summary, the reviewers support the approach of comparing EEG and FMRI using the same study and find the paper timely given the wealth of data linking theta oscillations to memory encoding. However, the statistical approach to the fMRI and EEG data are not sufficiently clear and need to be clarified. The concern is whether there is a single general trend in the dataset - whereby MTL activity (including theta power and BOLD activity) correlates with memory performance - and this single pattern is captured in both the spatial and memory memory tasks. It appear that the FMRI data were exposed to a 2x2 ANOVA, which sounds legitimate - but it is unclear if the EEG data were also analyzed in the same way, as compared to using t tests. Please clarify that the EEG data (and fMRI data) both convincingly show main effects of both performance and task.

I am also including the Reviewer's other comments below as it would be advisable to try to address the other concerns raised as well.

Rev 1:

On both the MRI and EEG data the authors provide two different statistical tests that they describe as being independent but that are not, for reasons I describe in my review below. I think this is a key problem.

I also am not sure that I am entirely convinced by their spatial localizations. I think they are doing excessive thresholding of their data to tell a story that the effect is localized to the MTL when in fact the effects are brain wide.

This manuscript examines fMRI and EEG recordings from subjects performing a memory task where they were instructed to use either a spatial or a non-spatial strategy on different trials. The data were analyzed to identify neural correlates of each strategy, as well as those related to memory encoding overall. The two main identified neural signals---main effects of spatial processing and successful memory encoding---were localized to the same brain region. The authors interpret the colocalization of these patterns as important because they provide an explanation for why spatial strategies improve memory, by boosting ongoing neural signals that are related to encoding more generally.

This is an interesting topic overall. However, I have critical concerns with their data analyses and interpretations. As I describe below, I see an alternate explanation for their results that they haven't ruled out and that is much less interesting than their primary view.

Primary comments:

* The paper's key point is that MTL theta oscillations and BOLD activity decrease in the same areas for spatial processing compared to non-spatial processing, as well as for successful compared to unsuccessful memory encoding. I believe there is a key confound here because the spatial and memory effects are analyzed in a way that they are not necessarily independent. Memory recall performance is significantly greater for spatial compared to non-strategies (Figure 1). This leaves open the possibility that the observed patterns are caused by neural signals correlated with performance, such as attention. My concern is that even if theta decreases (and BOLD activity) are related only to memory performance they could show up in the spatial vs. non-spatial contrast due to the sizable performance differences between these conditions that were not corrected.

To address this concern the authors need to show that the spatial versus non-spatial theta and BOLD effects are not explained by performance differences between spatial and non-spatial strategies. A general linear model is one method that the authors might consider, as this has been used previously to differentiate which of multiple interacting factors best explain a dependent variable.

* I have an additional concern about the source localization findings. The analyses of the raw scalp EEG data showed memory- and strategy-related theta power decreases across nearly the entire scalp (Fig. 3C). Thus, it seems to me that the effect is brain-wide. However, the authors discussed these results as if they were specific to the MTL, after processing with beamforming and a high degree of statistical thresholding (see Fig 4 left panels). My sense is that the authors claims of region specificity is not justified. If the authors really want to show that the theta results are specific to the hippocampus and MTL, then they need to show the magnitude of the effects in other areas like the neocortex and demonstrate that the results there differ significantly from the MTL.

Minor points:

* The findings would have been more interesting if the same subjects had participated in both EEG and MRI scanning, because this would have allowed the researchers to examine intersubject differences. Some of the manuscript misleadingly suggests that this was indeed the case, especially with the use of the word "co-occur" (including in the title). This issue could be corrected by replacing this word with a phrase like "occur in the same region as".

* In a related study, Scheeringa et al. (2009) found similar patterns of memory-related activations with simultaneous EEG and fMRI. The authors should compare their findings to this paper.

* I find it confusing that the direction of the theta MTL effects here flips compared to the Hanslmayr et al. (2011) study. I didn't find the text in the Discussion related to this issue to be especially compelling because it seems to simply dismiss the earlier study as having "weak" effects. Actually, the effects switch direction between these two studies. I would expect a stronger explanation for why an effect that is presumably related to memory would be robust in two studies but in opposite directions.

* The paper would be strengthened if authors could substantiate the use of beamforming scalp EEG data to estimate activity from deep brain structures. Has it been directly shown that beamforming of scalp EEG data provides an accurate measurement of MTL activity? My sense is that most beamforming validation work is done with MEG data.

Rev 2:

The authors present independent fMRI and EEG studies focussing on BOLD and theta increases/decreases during encoding, whilst participants use two well-known mnemonic strategies. They find MTL BOLD increases for method of loci vs pegword, and for subsequent remembered vs forgotten items in fMRI, and MTL theta decreases for the same contrasts in EEG. They conclude that MTL BOLD increases parallel MTL theta decreases during memory encoding.

The studies are interesting, and contribute to the field. In particular, the lack of consistent results with regard to theta increases/decreases during encoding in the literature is unresolved - and this study contributes to this debate (though does not solve the issue). I agree with many of the previous reviewers comments, and the authors appeared to have responded well to this previous round of reviews. I have a few comments of my own:

1. The general pattern in the EEG is theta decreases and beta increases. The authors do not seem to discuss the beta effect much (focussing on the alpha increase). I may be mistaken, but I thought that beta decrease SME were a common finding in the literature (I thought this was much more robust than the direction of theta effects), but here you see increases. In short, it seems the two frequency bands (theta and alpha/beta) have flipped in this study relative to previous ones. This makes me question the theta effects more, so perhaps commenting on this relationship would be beneficial.

2. The fMRI small-volume effects are localised to the parahippocampal gyrus, and no hippocampal effect is reported. This seems surprising given previous literature - why don't you see anything in the hippocampus? The authors accurately report the parahippocampal gyrus effect, but then go on to discuss the MTL in general. I think this is slightly misleading, and implicitly (to me at least) suggests the hippocampus is also involved.

Minor comments:

1. P. 16 - "These parallels are remarkable and indicate" - remarkable seems a bit strong here. Perhaps just "These parallels indicate".

2. P. 17 - "during spatial processing in the here reported datasets" - this sentence reads poorly.

3. Figure 7 - the fMRI SME effect does not appear to be present in the spatial condition in this graph. The figure legend doesn't explain how this ROI was identified, so perhaps a little more information is needed here.
